# A mutation in transmembrane protein 135 impairs lipid metabolism in mouse eyecups

**DOI:** 10.1038/s41598-021-04644-3

**Published:** 2022-01-14

**Authors:** Michael Landowski, Vijesh J. Bhute, Tetsuya Takimoto, Samuel Grindel, Pawan K. Shahi, Bikash R. Pattnaik, Sakae Ikeda, Akihiro Ikeda

**Affiliations:** 1 Department of Medical Genetics, University of Wisconsin-Madison, Madison, WI USA; 2 McPherson Eye Research Institute, University of Wisconsin-Madison, Madison, WI USA; 3 Department of Chemical Engineering, Imperial College London, South Kensington, London, SW7 2AZ UK; 4 Department of Ophthalmology and Visual Sciences, University of Wisconsin-Madison, Madison, WI USA

**Keywords:** Cell biology, Genetics, Molecular biology

## Abstract

Aging is a significant factor in the development of age-related diseases but how aging disrupts cellular homeostasis to cause age-related retinal disease is unknown. Here, we further our studies on transmembrane protein 135 (*Tmem135*), a gene involved in retinal aging, by examining the transcriptomic profiles of wild-type, heterozygous and homozygous *Tmem135* mutant posterior eyecup samples through RNA sequencing (RNA-Seq). We found significant gene expression changes in both heterozygous and homozygous *Tmem135* mutant mouse eyecups that correlate with visual function deficits. Further analysis revealed that expression of many genes involved in lipid metabolism are changed due to the *Tmem135* mutation. Consistent with these changes, we found increased lipid accumulation in mutant T*mem135* eyecup samples. Since mutant *Tmem135* mice have similar ocular pathologies as human age-related macular degeneration (AMD) eyes, we compared our homozygous *Tmem135* mutant eyecup RNA-Seq dataset with transcriptomic datasets of human AMD donor eyes. We found similar changes in genes involved in lipid metabolism between the homozygous *Tmem135* mutant eyecups and AMD donor eyes. Our study suggests that the *Tmem135* mutation affects lipid metabolism as similarly observed in human AMD eyes, thus *Tmem135* mutant mice can serve as a good model for the role of dysregulated lipid metabolism in AMD.

## Introduction

The most significant risk factor for age-related diseases is aging itself. However, how aging is associated with age-related disease at the molecular level is still largely to be understood. The polygenic nature of age-related neurodegenerative diseases such as age-related macular degeneration (AMD) and Alzheimer’s disease makes identification of the molecular pathways involved more challenging^1,2^.Therefore, mouse mutants showing age-related symptoms are valuable for isolating genetic factors that contribute to aging and age-related diseases.

We identified one such mouse strain harboring a mutation in transmembrane protein 135 (*Tmem135*^*FUN025/FUN025*^) that displays signs of accelerated aging in the retina as well as pathologies observed in AMD including autofluorescent aggregates in the subretinal space, increased inflammation, and thickened retinal pigmented epithelium (RPE) at a young age^3^. These phenotypes indicated that *Tmem135* regulates retinal aging and its impairment results in age-related disease phenotypes. *Tmem135* encodes a 52 kilodalton protein with five transmembrane domains that share similarities with the Tim17 protein family, a group of proteins important for mitochondria biogenesis^3,4^. TMEM135 was originally identified as a gene associated with longevity and cold stress in C. elegans^5^. Previous studies also suggested the function of TMEM135 in lipid metabolism^5,6^. In addition, a study using transgenic mice overexpressing the *Tmem135* gene (*Tmem135* TG) showed that *Tmem135* overexpression results in RPE dysmorphogenesis and degeneration^7^. Electron micrographs of the RPE from *Tmem135*^*FUN025/FUN025*^ mice showed over-fused (elongated) mitochondrial networks while the RPE of *Tmem135* TG mice displayed over-fragmented mitochondrial networks relative to controls^3,7^. Despite their morphological differences, both *Tmem135*^*FUN025/FUN025*^ and *Tmem135* TG RPE show reduced mitochondrial function^8^. Taken together, these results strongly indicated that TMEM135 acts as a pro-mitochondrial fission factor and regulates “mitochondrial dynamics,” the collective term for the fusion, fission, and mitophagy events mitochondria undergo to preserve their shape, number and function^9^, which is critical for RPE health. One striking age-dependent phenotypic feature of *Tmem135*^*FUN025/FUN025*^ mice is the robust infiltration of IBA1- and F4/80-positive immune cells into the subretinal space^3^. Accumulation of subretinal immune cells has been associated with all stages of AMD and believed to contribute to the chronic inflammation underlying AMD. The phenotypic similarity between *Tmem135*^*FUN025/FUN025*^ mice and human AMD motivates us to identify molecular pathways underlying the retinal pathologies in *Tmem135*^*FUN025/FUN025*^ mice.

In this study, we performed RNA sequencing (RNA-Seq) of the eyecup (RPE/choroid/sclera) of *Tmem135*^*FUN025*^^*/FUN025*^ mice compared with heterozygous and wild-type (WT) mice. We found several pathways including cholesterol metabolism, fatty acid metabolism, and steroid metabolic processes were significantly upregulated in *Tmem135*^*FUN025/FUN025*^ mice. We observed increased lipid accumulations in *Tmem135*^*FUN025/FUN025*^ eyecups, which confirms the changes in lipid metabolism. Some of these gene expression changes overlap with those previously observed in human AMD-afflicted RPE/choroid samples. Our study suggests that lipid metabolism is a molecular pathway affected by the *Tmem135* mutation as observed in human AMD eyes, thus *Tmem135* mutant mice can serve as a good model for the role of dysregulated lipid metabolism in AMD.

## Results

### Heterozygous and homozygous Tmem135 mutant mice display ocular phenotypes

**Figure 1:**
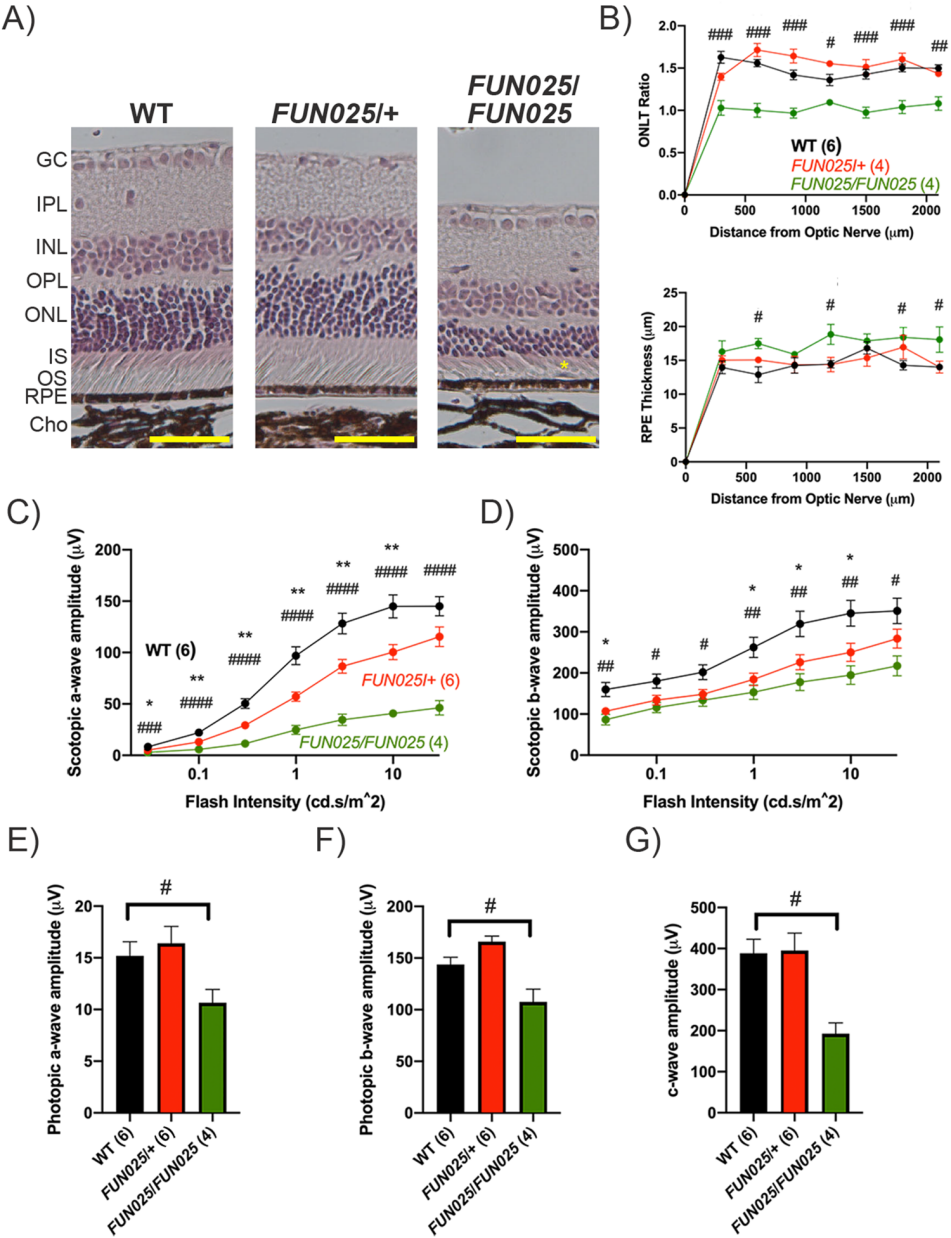
Retinal Phenotype of 12-Month-Old *Tmem135*^*FUN025*^^*/*+^ mice. (**A**) Representative images of the retinas of 12-month-old WT, *Tmem135*^*FUN025/*+^ (*FUN025/*+*)* and *Tmem135*^*FUN025/FUN025*^ (*FUN025/FUN025*) mice. Note the decreased ONL thickness and subretinal immune cell (denoted with a yellow asterisk) in the 12-month-old *Tmem135*^*FUN025/FUN025*^ retina which are not present in the 12-month-old *Tmem135*^*FUN025/*+^ mice. Magnification = 20×. Scale bar = 100 microns. (**B**) ONLT and RPE ratios. No significant differences were noted between the WT and *Tmem135*^*FUN025/*+^ mice. (**C-G**) ERG recordings for 12-month-old WT, *Tmem135*^*FUN025/*+^ and *Tmem135*^*FUN025/FUN025*^ mice during scotopic and photopic conditions. ^*^ and ^**^ indicates a P < 0.05 and *P* < 0.01 significance by post hoc Tukey test following a significant difference detected by one-way ANOVA between WT and *Tmem135*^*FUN025/*+^. ^#^, ^##^, ^###^, and ^####^ indicates a P < 0.05, P < 0.01, P < 0.001, and *P* < 0.0001significance by post hoc Tukey test following a significant difference detected by one-way ANOVA between WT and *Tmem135*^*FUN025/FUN025*^ mice. All data are mean ± s.e.m.

A mutation in *Tmem135* effects retinal homeostasis in mice^3,7^. Homozygous *Tmem135* mutant (*Tmem135*^*FUN025*^^/*FUN025*^) mice show early-onset of aging-associated retinal changes with accelerated progression and pathologies similar to those observed in AMD as early as 2 months of age^3^. At that age, heterozygous *Tmem135* mutant (*Tmem135*^*FUN025*/+^) mice did not show any ocular abnormalities (data not shown). Here, we investigated whether heterozygous *Tmem135* mutant (*Tmem135*^*FUN025*/+^) mice display ocular abnormalities at 12 months of age. No distinct morphological changes were observed in *Tmem135*^*FUN025*/+^ retinal sections compared to WT retinal sections (Fig. 1A). This contrasts the presence of photoreceptor degeneration and subretinal immune cells present in homozygous *Tmem135* mutant (*Tmem135*^*FUN025*/*FUN025*^) retinas (Fig. 1A). We quantitated photoreceptor degeneration in *Tmem135*^*FUN025*/*FUN025*^ retinas by normalizing the length of the outer nuclear layer (ONL) to the inner nuclear layer (INL) to calculate the ONL thickness (ONLT) ratio^3^. While there were no ONLT differences between WT and *Tmem135*^*FUN025*/+^, the ONLT ratios of both groups were significantly higher than those measured for the *Tmem135*^*FUN025*/*FUN025*^ retinas (Fig. 1B). Also, there were no differences in the thickness of WT and *Tmem135*^*FUN025*/+^ RPE, which is increased in *Tmem135*^*FUN025*/*FUN025*^ mice (Fig. 1B). Next, we measured visual function through electroretinography (ERG). 12-month-old *Tmem135*^*FUN025*/+^ mice had significantly smaller scotopic a-waves than WT mice except at the 30 cd*s/m^2^ flash intensity (Fig. 1C). The scotopic b-waves of *Tmem135*^*FUN025*/+^ mice were significantly decreased at 0.03, 1, 3, and 10 cd*s/m^2^ flash intensities relative to WT mice (Fig. 1D). No changes in the photopic a-wave, photopic b-wave, and c-wave were noted between WT and *Tmem135*^*FUN025*/+^ mice (1E-G). Given that scotopic ERG responses and not photopic ERG responses were changed, rods are sensitive to the heterozygosity of the *Tmem135* mutation. The scotopic a-waves and b-waves at all intensities as well as photopic a-wave, photopic b-wave, and c-wave were significantly attenuated between *Tmem135*^*FUN025*/*FUN025*^ and WT mice (Fig. 1C-G) as previously described^3^. Our data indicates both *Tmem135*^*FUN025*/+^ and *Tmem135*^*FUN025*/*FUN025*^ mice have retinal functional abnormalities by 12 months of age, but only *Tmem135*^*FUN025*/*FUN025*^ mice have pronounced retinal morphological differences compared to WT mice.

### Mutation in Tmem135 results in widespread transcriptional changes

**Figure 2:**
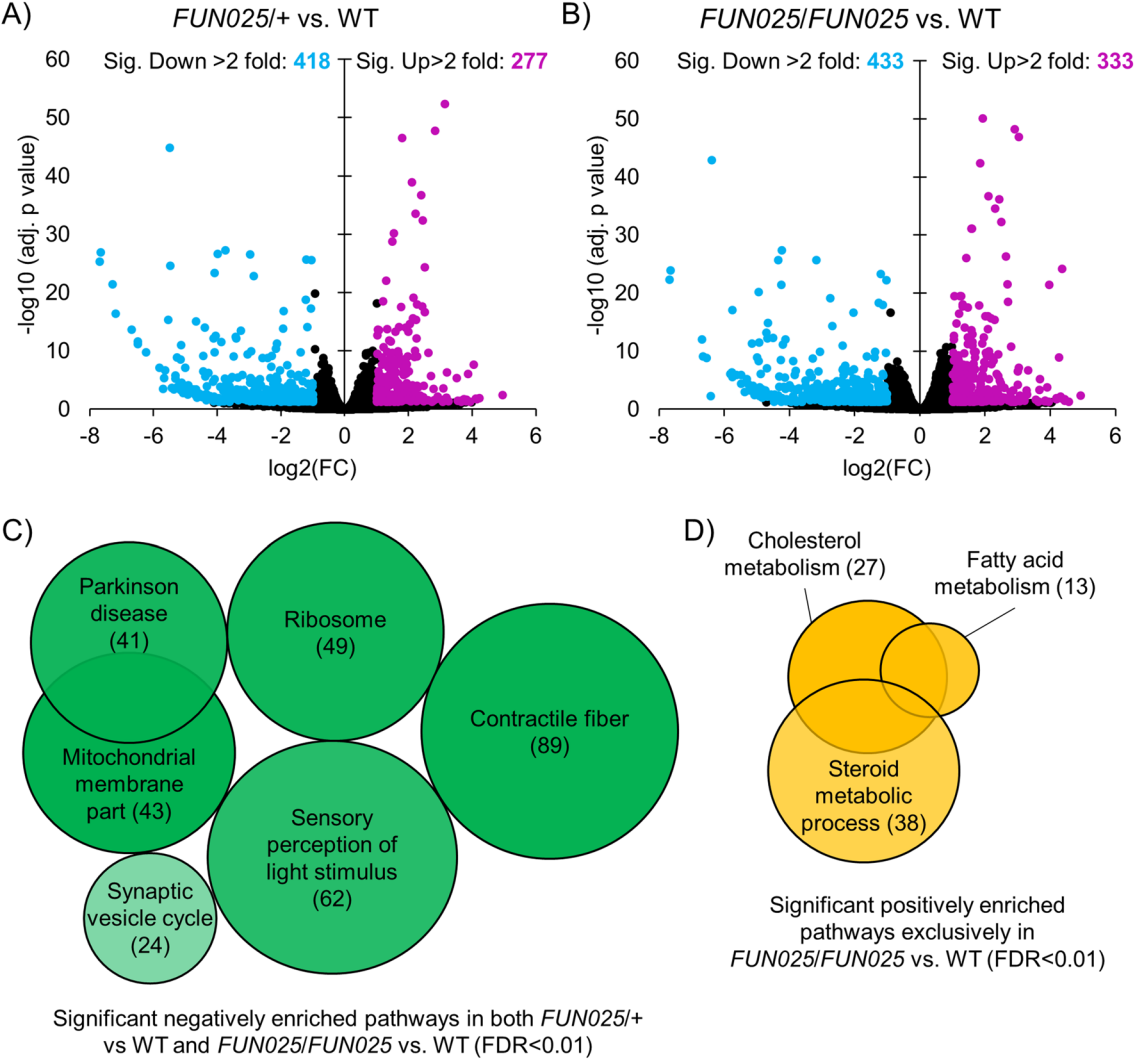
RNA-Seq analysis of 2.5-month-old heterozygous and homozygous *Tmem135* mutant eyecups compared to WT eyecups. Volcano plot highlighting differentially-expressed genes with fold changes greater than 2 that reach statistical significance (*p* < 0.05) between (**A**) *Tmem135*^*FUN025*^^/+^ (*FUN025/*+*)* and WT eyecups as well as (**B**) *Tmem135*^*FUN025/FUN025*^ (*FUN025/FUN025*) and WT eyecups. Genes that are upregulated and downregulated are highlighted in magenta and cyan, respectively. Gene Set Enrichment Analysis (GSEA) results of significantly enriched gene sets (FDR < 0.01) of (**C**) downregulated genes and (**D**) upregulated in *Tmem135*^*FUN025*/+^ and *Tmem135*^*FUN025/FUN025*^ eyecups compared to WT eyecups. The numbers within brackets indicate the number of genes in the leading edge in the gene set and the colour indicates the normalized enrichment score where the lighter tone means less enrichment and darker tone means more enrichment.

To identify the origin of the retinal abnormalities due to the *Tmem135* mutation, we performed RNA-Seq on eyecups collected from 2.5-month-old WT, *Tmem135*^*FUN025*^^/+^, and *Tmem135*^*FUN025*/*FUN025*^ mice. This time point was selected in order to examine transcriptional changes in the early stage of the pathological changes in *Tmem135*^*FUN025*/*FUN025*^ mice rather than later stages that would be affected by extensive retinal degeneration (Fig. 1)^3^. These eyecup tissue preparations include the retinal pigmented epithelium (RPE), a cell-type particularly sensitive to the levels of functional TMEM135^7^ and postulated as the primary site affected by the *Tmem135*^*FUN025*^mutation^3^. Global transcription profiles were affected in both *Tmem135*^*FUN025*/+^ and *Tmem135*^*FUN025*/*FUN025*^ eyecups compared with WT controls. 1723 genes and 1785 genes were significantly altered (adj.p value < 0.05) in the *Tmem135*^*FUN025*/+^ and *Tmem135*^*FUN025*/*FUN025*^ genotypes relative to WT, respectively. 1112 were commonly affected between the *Tmem135*^*FUN025*/+^ and *Tmem135*^*FUN025*/*FUN025*^ eyecups compared to WT. There were 611 and 673 genes uniquely changed in *Tmem135*^*FUN025*/+^ and *Tmem135*^*FUN025*/*FUN025*^ eyecups, respectively. We narrowed our RNA-seq analysis on genes with twofold expression differences in *Tmem135*^*FUN025*/+^ and *Tmem135*^*FUN025*/*FUN025*^ eyecups (Fig. 2A,B). In *Tmem135*^*FUN025*/+^ eyecups, there were 277 and 418 genes that had twofold upregulation and downregulation compared to WT, respectively (Fig. 2A). Similarly, there were 333 genes that had twofold upregulation and 433 genes that had twofold downregulation in *Tmem135*^*FUN025*/*FUN025*^ eyecups (Fig. 2B).

Next, we conducted gene set enrichment analysis (GSEA) to identify potential pathways which are affected due to the *Tmem135* mutation. Several pathways were significantly downregulated (FDR < 0.01) in both *Tmem135*^*FUN025*^^/+^ and *Tmem135*^*FUN025*/*FUN025*^ as compared to WT including contractile fiber, sensory perception of light stimulus, ribosome, mitochondrial membrane part, Parkinson disease, and synaptic vesicle cycle (Table 1 and Fig. 2C). On the other hand, several pathways including cholesterol metabolism, fatty acid metabolism, and steroid metabolic processes were significantly upregulated (FDR < 0.01) exclusively in *Tmem135*^*FUN025*/*FUN025*^ eyecups relative to WT (Table 1 and Fig. 2D). Importantly, none of the pathways were significantly upregulated (FDR < 0.01) between *Tmem135*^*FUN025*/+^ and WT eyecups (Table 1). Our results show there are distinct pathways disrupted by the *Tmem135* mutation in the murine eyecup.

**Table 1:** Pathway analysis using Gene Set Enrichment Analysis on *Tmem135*^*FUN025*^^/+^ and *Tmem135*^*FUN025*/*FUN025*^ compared to WT gene sets*.

**Description**	***Tmem135***^***FUN025***^^**/+**^** vs WT**		***Tmem135***^***FUN025***^^**/*FUN025***^** vs WT**	
	**NES**	**FDR**	**NES**	**FDR**
WP4346: Cholesterol metabolism			2.71	0
mmu01212: Fatty acid metabolism			2.53	0
GO:0,008,202: Steroid metabolic process			2.33	0.0023
mmu04721: Synaptic vesicle cycle	− 1.78	0.0225	− 1.88	0.0058
GO:0,050,953: Sensory perception of light stimulus	− 2.11	0.0009	− 1.97	0.0141
mmu05012: Parkinson disease	− 2.17	0	− 2.01	0.0012
mmu03010: Ribosome	− 2.21	0	− 2.03	0.0018
GO:0,043,292: Contractile fiber	− 2.22	0	− 2.06	0
GO:0,044,455: Mitochondrial membrane part	− 2.13	0	− 2.1	0

*FDR cut-off of 0.01 is used for analysis. Empty cells indicate FDR was greater than 0.01.

### Transcriptomic differences between heterozygous and homozygous Tmem135 mutant mice

**Figure 3:**
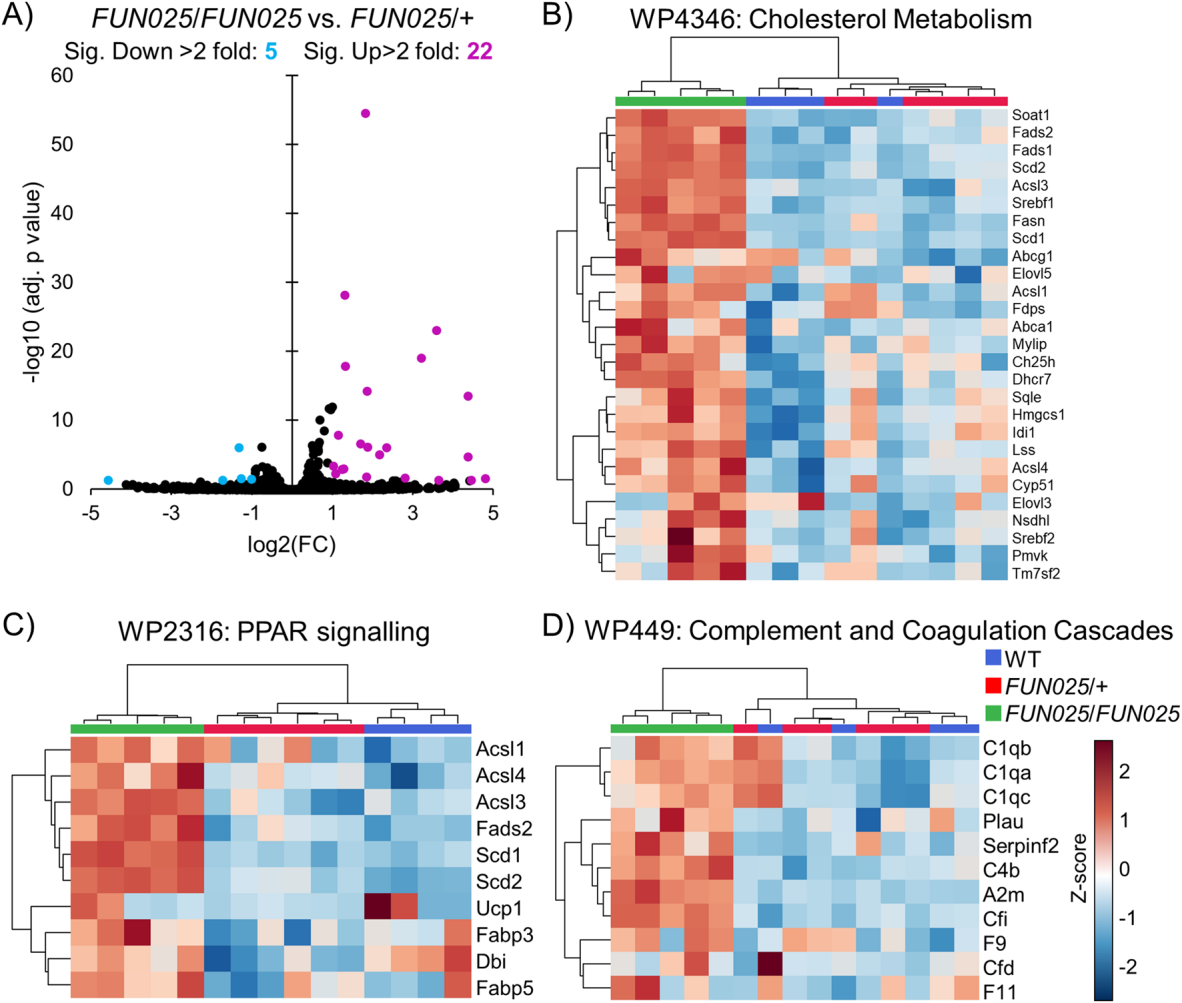
RNA-Seq analysis of 2.5-month-old homozygous *Tmem135* mutant eyecups compared to heterozygous *Tmem135* mutant eyecups. (**A**) Volcano plot highlighting differentially-expressed genes with fold changes greater than 2 that reach statistical significance (*p* < 0.05) between *Tmem135*^*FUN025/FUN025*^ (*FUN025/FUN025*) and *Tmem135*^*FUN025/*+^ (*FUN025*/+) eyecups. Genes that are upregulated and downregulated are highlighted in magenta and cyan, respectively. Heatmaps of differentially-expressed genes in the leading edge with greater than log2 fold changes between *Tmem135*^*FUN025/FUN025*^ and *Tmem135*^*FUN025/*+^ eyecups involved in (**B**) cholesterol metabolism, (**C**) PPAR signalling, and (**D**) complement and coagulation cascades which were identified using gene set enrichment analysis. These genes were unchanged between *Tmem135*^*FUN025/*+^ and WT eyecups.

To further elucidate important pathways perturbed by the *Tmem135* mutation, we undertook a differential analysis focusing on the *Tmem135*^*FUN025*^^/+^ and *Tmem135*^*FUN025*/*FUN025*^ genotypes. There were 84 genes which showed significant differences (adj. *p* < 0.05), and 27 of these genes had more than two-fold expression changes. 5 of these genes had twofold decreased expression whereas 22 genes had twofold increased expression in *Tmem135*^*FUN025*/+^ eyecups relative to *Tmem135*^*FUN025*/*FUN025*^ eyecups (Fig. 3A). We performed GSEA to identify pathways enriched in *Tmem135*^*FUN025*/*FUN025*^ eyecups relative to *Tmem135*^*FUN025*/+^ eyecups. There was significant enrichment of cholesterol metabolism (NES = 2.32, FDR = 0.013) (Fig. 3B), peroxisome proliferator-activated receptor (PPAR) signalling (NES = 2.28, FDR = 0.011) (Fig. 3C), and complement and coagulation cascades (NES = 2.18, FDR = 0.04) (Fig. 3D) in *Tmem135*^*FUN025*/*FUN025*^ eyecups when compared with *Tmem135*^*FUN025*/+^ eyecups. These genes were unchanged between *Tmem135*^*FUN025*/+^ and WT eyecups (Fig. 3B,D). Our results implicate cholesterol metabolism, PPAR signalling and complement and coagulation cascades as important pathways underlying the pathology development in *Tmem135*^*FUN025*/*FUN025*^ mice.

### Correlations between differential gene expression and number of Tmem135 mutant alleles

**Figure 4:**
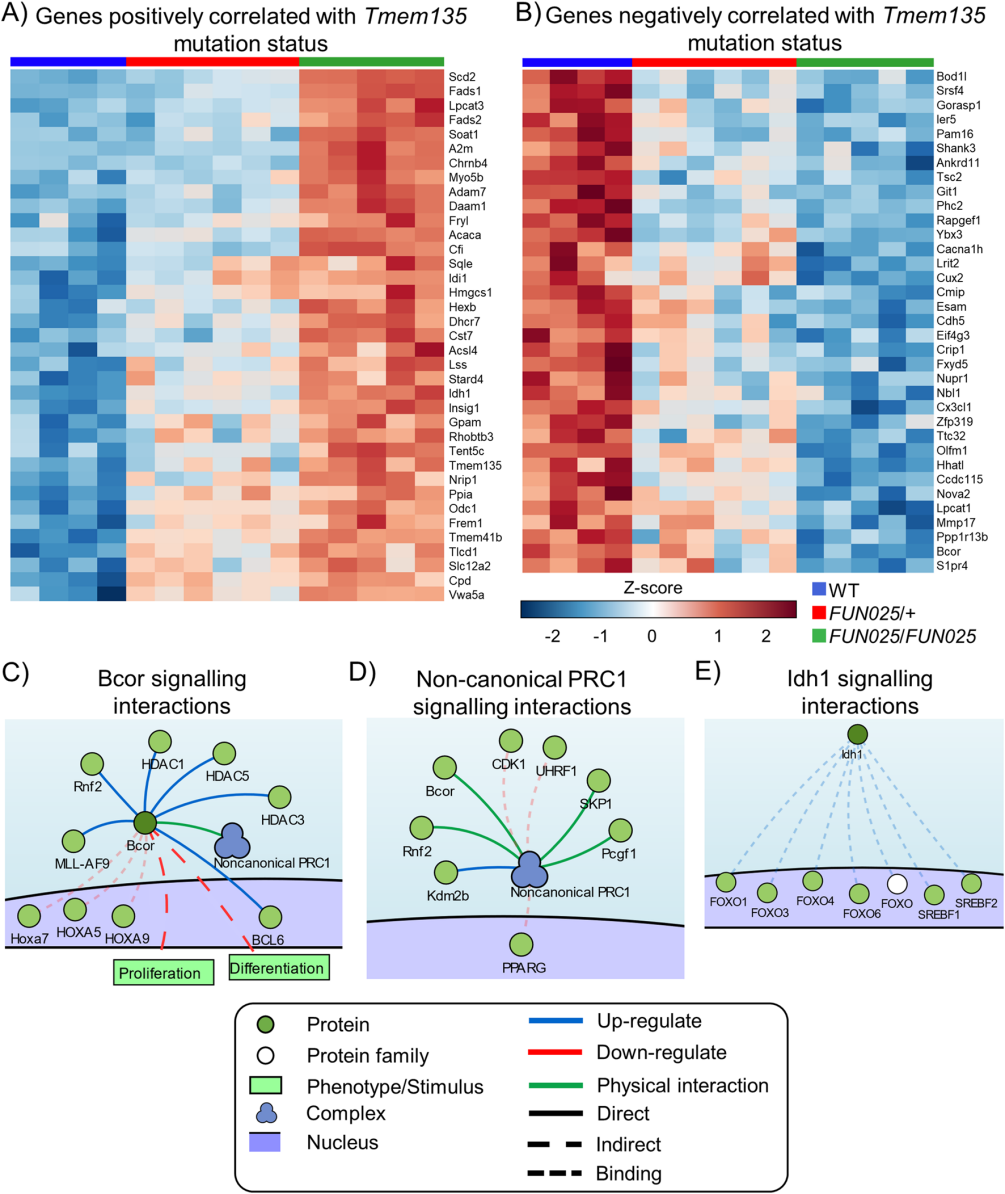
Correlation analysis highlights important signalling pathways affected by *Tmem135* mutations. Heatmap of genes which are significantly (**A**) positively and (**B**) negatively correlated (FDR < 0.01) with the number of mutant *Tmem135* alleles in 2.5-month-old WT, *Tmem135*^*FUN025*^^*/*+^, and *Tmem135*^*FUN025/FUN025*^ eyecups. Signalling network highlights interactions of (**C**) BCL6 corepressor (*Bcor*), (**D**) non-canonical polycomb recessive complex 1 (PRC1), and (**E**) isocitrate dehydrogenase 1 (*Idh1*). Network generated using the SIGnalling Network Open Resource (SIGNOR 2.0).

We determined if any of the genes with differential expression correlated with the number of *Tmem135* mutant alleles. 72 genes had significant correlations (FDR < 0.01) with the *Tmem135* mutation status of mice where 35 genes were downregulated and 37 genes were upregulated in *Tmem135*^*FUN025*^^/*FUN025*^ eyecups when compared with WT eyecups (Fig. 4A, B). We used signalling network analysis to identify potential mechanisms which might be affected in a mutation dependent manner. We identified BCL6 corepressor (*Bcor*) which had a negative correlation score of -0.9. BCOR can repress the transcription of target genes by interacting with histone deacetylases (HDACs) (Fig. 4C) and non-canonical polycomb recessive complex 1 (PRC1) that can downregulate peroxisome proliferator activated receptor gamma (*Pparg*) expression (Fig. 4D). We also observed a very strong positive correlation of 0.98 for isocitrate dehydrogenase 1 (*Idh1*) which was significantly increased in *Tmem135*^*FUN025*/*FUN025*^ mice. *Idh1* expression can be regulated by FOXO transcription factors and sterol regulatory element binding proteins (SREBFs) (Fig. 4E), both of which play important roles in lipid metabolism^10,11^. This analysis reveals a set of genes whose expression is correlated with the amount of functional TMEM135 and connected with transcriptomic pathways disrupted by the *Tmem135* mutation.

### Lipid levels in heterozygous and homozygous Tmem135 mutant mice

**Figure 5:**
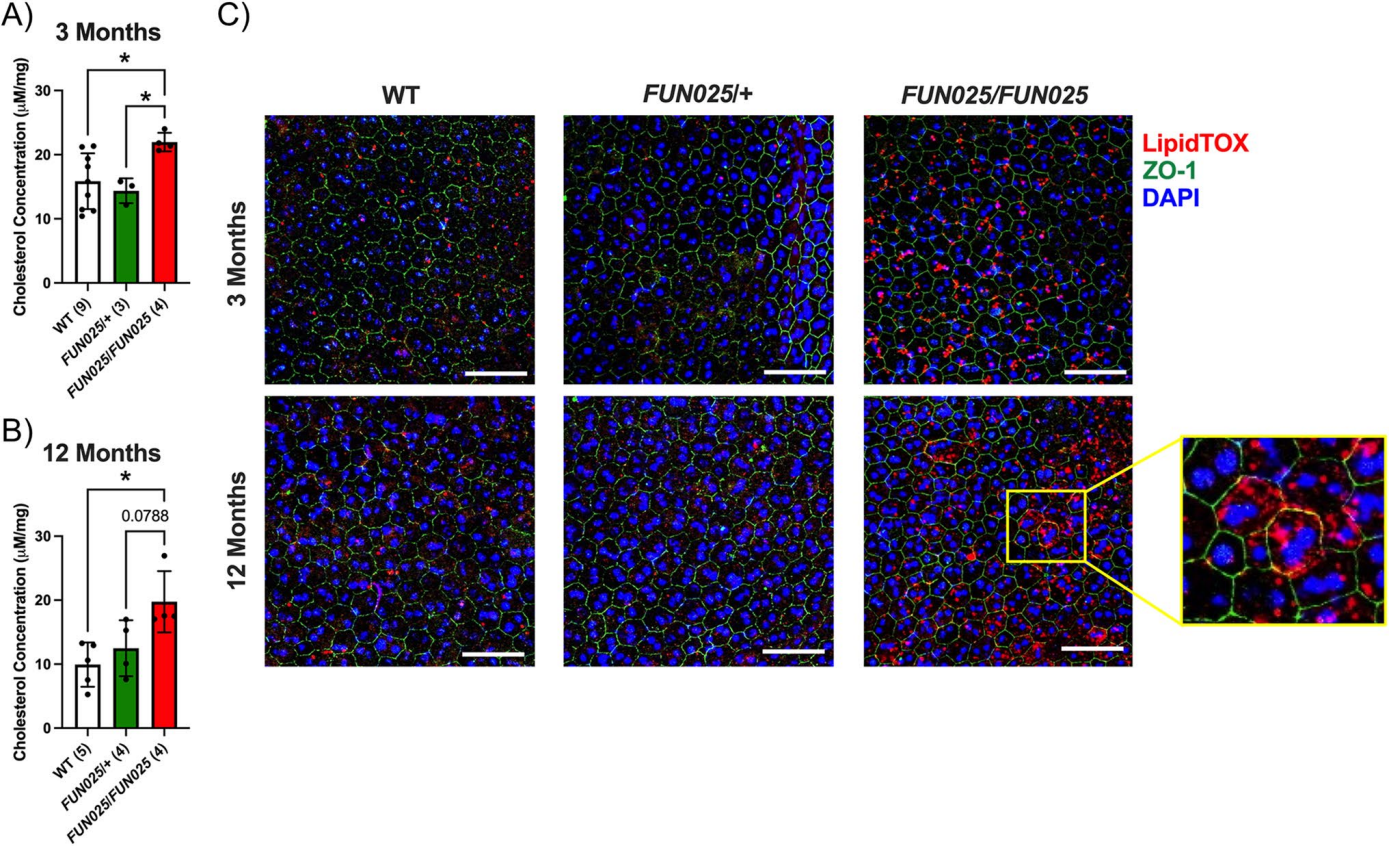
Lipid Levels in WT, Heterozygous and Homozygous *Tmem135* Mutant Eyecups. Cholesterol levels in eyecups of (**A**) 3-month-old and (**B**) 12-month-old WT, *Tmem135*^*FUN025*^^*/*+^ (*FUN025/* +)*,* and *Tmem135*^*FUN025/FUN025*^ (*FUN025/FUN025*) mice. * indicates a *P* < 0.05 significance by post hoc Tukey test following a significant difference detected by one-way ANOVA. Numbers with brackets denote number of mice used for experiment. Each dot represents each individual sample. Data is presented as mean ± s.d.. (**C**) Representative RPE flat mount images after labelling for tight junctions (green) and staining for neutral lipids (red) and nuclei (blue). Mag = 20×, Scale Bar = 25 microns.

To validate our RNA-Seq results, we determined lipid levels within the eyecups of *Tmem135*^*FUN025*^^/+^ and *Tmem135*^*FUN025*/*FUN025*^ mice. We prepared eyecup samples from 3-month-old and 12-month-old WT, *Tmem135*^*FUN025*/+^, and *Tmem135*^*FUN025*/*FUN025*^ mice to measure cholesterol concentrations. We detected significant increases of cholesterol in 3-month-old *Tmem135*^*FUN025*/*FUN025*^ eyecups (21.97 ± 1.45 μM/mg) compared to WT (15.85 ± 4.35 μM/mg) and *Tmem135*^*FUN025*/+^ eyecups (14.37 ± 1.94 μM/mg) (Fig. 5A). Similar increases in cholesterol were observed in 12-month-old *Tmem135*^*FUN025*/*FUN025*^ eyecups (19.76 ± 4.79 μM/mg) relative to WT (9.92 ± 3.46 μM/mg) and *Tmem135*^*FUN025*/+^ (12.49 ± 4.38 μM/mg) (Fig. 5B). We prepared RPE flat mounts from 3-month-old and 12-month-old WT, *Tmem135*^*FUN025*/+^, and *Tmem135*^*FUN025*/*FUN025*^ mice for staining with HSC LipidTox Red that detects neutral lipids and allows for their visualization. We detected an age-dependent increase of neutral lipid accumulations in *Tmem135*^*FUN025*/*FUN025*^ RPE flat mounts compared to WT and *Tmem135*^*FUN025*/+^ flat mounts (Fig. 5C). Together, these data support the increased expression of genes involved in lipid metabolism by showing increased lipid accumulations in *Tmem135*^*FUN025*/*FUN025*^ eyecups.

### Lipid metabolic gene expression in eyecups and neural retinas

**Figure 6:**
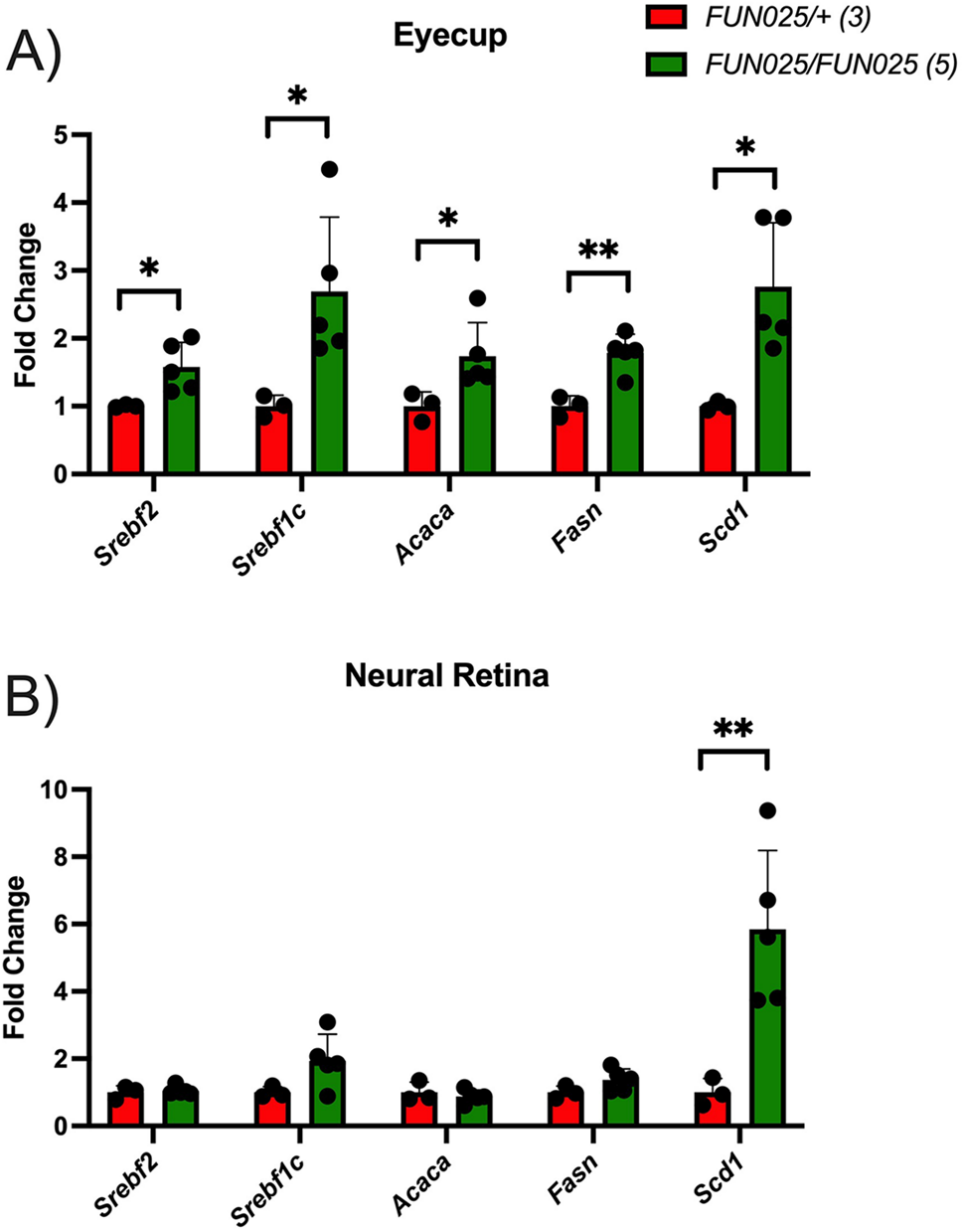
Lipid Metabolic Gene Expression in Eyecup and Neural Retinas of 2.5-month-old Heterozygous and Homozygous *Tmem135* Mutant Mice. Quantitative PCR analysis of lipid metabolic transcription factors (*Srebf2* and *Srebf1c*) and lipid synthesis genes (*Acaca*, *Fasn*, and *Scd1*) in the (**A**) eyecups and (**B**) neural retinas of 2.5-month-old male *Tmem135*^*FUN025*^^*/*+^ (*FUN025/* +*)* and *Tmem135*^*FUN025/FUN025*^ (*FUN025/FUN025*) mice. Numbers in brackets denote number of mice used in experiment. Each dot represents each individual sample. * and ** indicates a *P* < 0.05 and *P* < 0.01 significance by Student’s T-test.

The RPE is a major tissue involved in lipid metabolism within the retina^12^. Since we detected significant lipid metabolic transcriptional changes from our RNA-seq study, we explored whether these changes were unique to the posterior eyecup containing the RPE. We prepared cDNA from RNA individually isolated from the posterior eyecup and neural retinas of 2.5-month-old *Tmem135*^*FUN025*^^/+^ and *Tmem135*^*FUN025*/*FUN025*^ mice for quantitative PCR (qPCR). We quantified sterol regulatory element binding transcription factor 2 (*Srebf2*), *Srebf1c*, acetyl-CoA carboxylase alpha (*Acaca*), fatty acid synthase (*Fasn*), and stearoyl-CoA desaturase 1 (*Scd1*). We found significant increases in all these genes in *Tmem135*^*FUN025*/*FUN025*^ eyecups compared to *Tmem135*^*FUN025*/+^ eyecups (Fig. 6A). Strikingly, only *Scd1* was upregulated in the neural retinas of *Tmem135*^*FUN025*/*FUN025*^ mice relative to *Tmem135*^*FUN025*/+^ neural retinas (Fig. 6B). These data show that *Tmem135* mutant-induced increases of lipid metabolic genes are predominantly confined to the posterior eyecup.

### Transcriptomic similarities between homozygous Tmem135 mutant eyecups and AMD donor eyes

**Figure 7:**
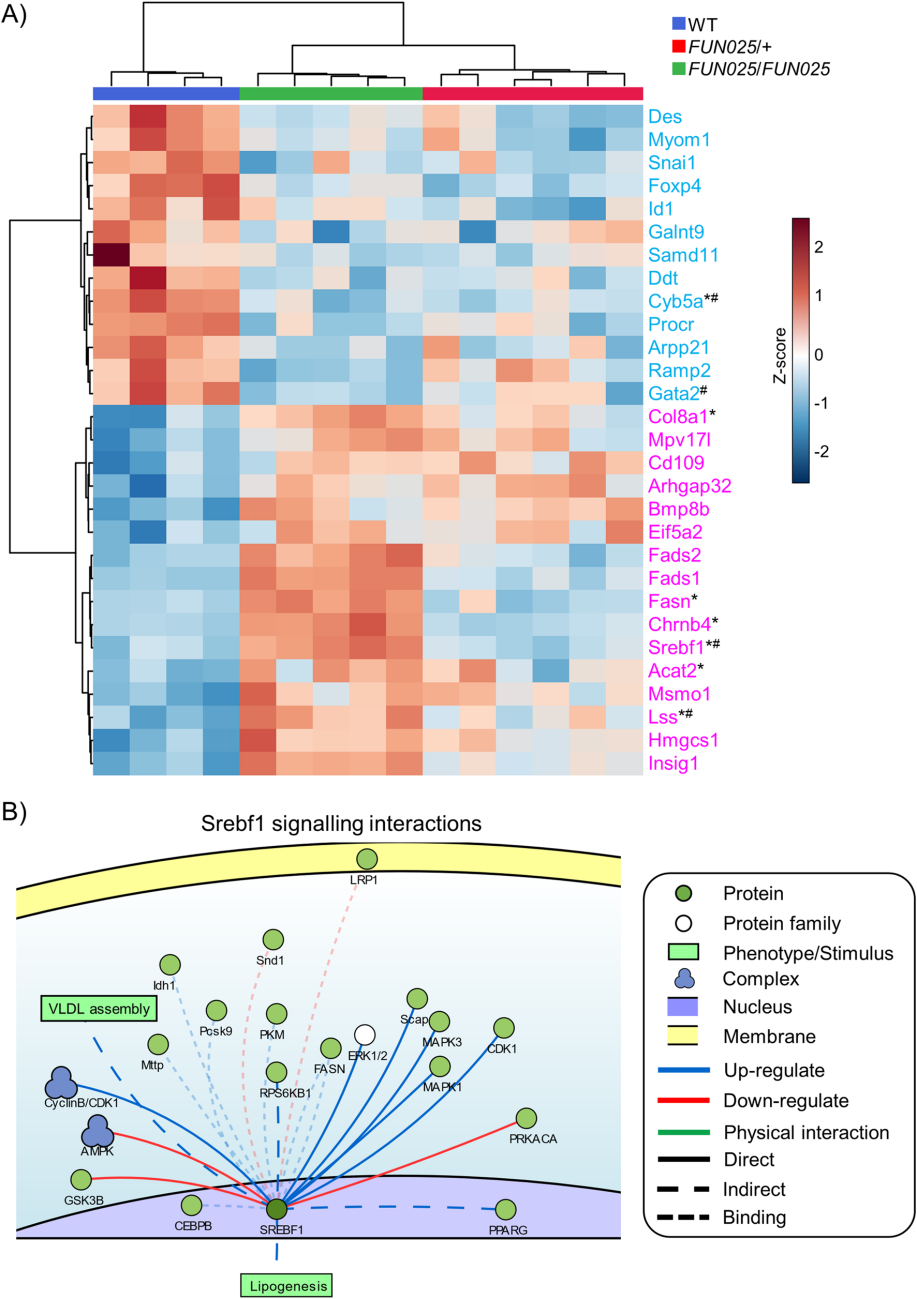
Comparison of *Tmem135*^*FUN025/FUN025*^ eyecup and AMD-afflicted RPE/choroid transcriptional profiles. (**A**) Heatmap showing the significantly altered genes from *Tmem135*^*FUN025/FUN025*^ vs. WT which are affected in a similar direction (up [magenta] or down [cyan]) when compared with integrated expression analysis from microarray and RNA-Seq data (GSE29801 and GSE135092) from AMD patients. Genes which are affected in *intermediate and ^#^advanced stage of AMD are also highlighted. (**B**) Signalling network highlights interactions of Sterol Regulatory Element Binding Transcription Factor* 1* (SREBF1). Network generated using the SIGnalling Network Open Resource (SIGNOR 2.0).

It is well accepted that dysfunction within the RPE is implicated as a key pathobiological mechanism in AMD^13–15^. Since the ocular phenotype of *Tmem135*^*FUN025*^^/*FUN025*^ mice shares similarities with AMD including the presence of subretinal immune cells, neuroinflammation and RPE autofluorescence^3,16^, we were interested in evaluating the eyecup transcriptional profiles of the *Tmem135*^*FUN025/FUN025*^ mice with those obtained from RPE/choroid samples from AMD patients. We compared our *Tmem135*^*FUN025/FUN025*^ versus WT RNA-Seq dataset with previously-published integrated microarray and RNA-Seq datasets (GSE29801 and GSE135092) using RPE/choroid samples from AMD patients^17–19^. There were similar genes affected due to the *Tmem135* mutation and AMD (Table 2), including 16 upregulated and 9 downregulated genes in common between the *Tmem135*^*FUN025*/*FUN025*^ relative to WT RNA-Seq dataset and GSE29801 and GSE135092 datasets (Table 2 and Fig. 7A). In the GSE29801 dataset, some AMD RPE/choroid samples were classified as having an intermediate and advanced AMD diagnosis^17^, allowing us to compare our *Tmem135*^*FUN025/FUN025*^ versus WT RNA-Seq dataset to specific stages of AMD. Six upegulated genes [cholinergic receptor nicotinic beta 4 subunit (*Chrnb4*), *Srebf1*, *Fasn*, acetyl-CoA acetyltransferase 2 (*Acat2*), collagen type VIII alpha 1 chain (*Col8a1*), and lanosterol synthase (*Lss*)] and one downregulated gene [cytochrome B5 type a (*Cyb5a*)] were common between *Tmem135*^*FUN025/FUN025*^ eyecups and intermediate AMD (Table 2 and Fig. 7A). Furthermore, two upregulated genes (*Srebf1* and *Lss*) and two downregulated genes [GATA binding protein 2 (*Gata2*) and *Cyb5a*] were common between *Tmem135*^*FUN025/FUN025*^ eyecups and advanced AMD (Table 2 and Fig. 7A). We performed a gene over-representation analysis on the genes in Table 2 and found significant enrichment (FDR < 0.01) of multiple lipid metabolic pathways including cholesterol metabolism, sterol biosynthetic process, sterol metabolic process, lipid biosynthetic process, fatty acid biosynthetic process, and fatty acid metabolism (Table 3). We also investigated the network signaling pathways commonly affected between *Tmem135*^*FUN025/FUN025*^ mice and AMD. We identified the SREBF1-signaling pathway, a pathway that mediates lipid homeostasis by regulating *Pparg* expression and lipogenesis (Fig. 7B)^11^, in common between *Tmem135*^*FUN025/FUN025*^ eyecups and AMD. Similarities between the *Tmem135*^*FUN025/FUN025*^ mice and AMD transcriptional profiles suggest the *Tmem135*^*FUN025*/*FUN025*^ mouse may serve as a valuable model to interrogate the role of lipid metabolism on AMD-like pathology development.

**Table 2:** Summary of significantly-affected genes in common between *Tmem135*^*FUN025/FUN025*^ versus WT eyecup RNAseq and GSE29801 and GSE135092 datasets.

Stage of AMD	Genes in Common Between *Tmem135*^*FUN025/FUN025*^ eyecups and AMD-afflicted RPE/choroid samples	
	Downregulated	Upregulated
Mixed	*Samd11, Gata2, Ddt, Cyb5a, Des, Procr, Galnt9, Arpp21, Ramp2*	*Chrnb4, Srebf1, Fasn, Acat2, Fads1, Fads2, Col8a1, Lss, Cd109, Bmp8b, Hmgcs1, Msmo1, Arhgap32, Mpv17l, Eif5a2, Insig1*
Intermediate	*Cyb5a*	*Chrnb4, Srebf1, Fasn, Acat2, Col8a1, Lss*
Advanced	*Gata2, Cyb5a*	*Srebf1, Lss*

**Table 3:** Pathway analysis using overrepresentation analysis (ORA) on genes significantly-affected in both *Tmem135*^*FUN025/FUN025*^ versus WT eyecup RNA-Seq and GSE29801 and GSE135092 datasets*.

Description	Enrichment ratio	FDR
WP4346: Cholesterol metabolism	26.40	7.41 × 10^–7^
GO:0016126: Sterol biosynthetic process	37.99	1.80 × 10^–3^
GO:0016125: Sterol metabolic process	18.16	1.81 × 10^–3^
GO:0008610: Lipid biosynthetic process	7.16	1.81 × 10^–3^
GO:0006633: Fatty acid biosynthetic process	17.35	1.81 × 10^–3^
mmu01212: Fatty acid metabolism	21.95	9.41 × 10^–3^

*FDR cut-off of 0.01 is used for analysis.

## Discussion

Our current study builds on previous work that identified TMEM135 as a key protein involved in mitochondrial dynamics^3,7,20,21^, metabolic regulation^8^ and oxidative stress in mice^3^. However, the molecular function of TMEM135 remains elusive. In our study, we evaluated the global transcriptome of posterior eyecups to understand how a *Tmem135* mutation disrupts retinal homeostasis in mice. Both *Tmem135*^*FUN025*^^/+^ and *Tmem135*^*FUN025*/*FUN025*^ mice have visual deficits such as diminished ERG responses although only *Tmem135*^*FUN025*/*FUN025*^ mice have structural abnormalities including photoreceptor degeneration and subretinal immune cell infiltration. We also found the eyecup transcriptional profiles of both *Tmem135*^*FUN025*/+^ and *Tmem135*^*FUN025*/*FUN025*^ mice deviate from WT controls, indicating one allele of the *Tmem135* mutation is sufficient to disrupt murine retinal homeostasis. Together, we conclude *Tmem135* is a critical gene involved in ocular health.

*Tmem135* encodes a membrane-bound protein that can localize with mitochondria^3,5^, peroxisomes^22^, and lipid droplets^5^. Our gene enrichment analysis highlighted significant downregulation of mitochondrial membrane and upregulation of lipid metabolism pathways in *Tmem135*^*FUN025*^^/*FUN025*^ eyecups. Mitochondria, peroxisomes, and lipid droplets interact with each other to maintain lipid metabolism within a cell^23^. In particular, cells depend on mitochondria for the complete breakdown of lipids through oxidative phosphorylation^24^. The mitochondria in *Tmem135*^*FUN025*/*FUN025*^ RPE appear overly elongated^7^ and correlates with increased levels of glycerol^8^, a building block of triglycerides. Triglycerides store fatty acids within the cell for consumption by mitochondria for energy utilization^25^. It has been shown that mitochondria with close proximities to lipid droplets are overly elongated^26^, which could explain the mitochondrial morphological changes in *Tmem135*^*FUN025*/*FUN025*^ RPE. Thus, TMEM135 may have a role in lipid metabolism within the RPE, supporting other studies linking the function of TMEM135 to lipid metabolism^5,6^.

The RPE is critical for sustaining lipid homeostasis within the retina, digesting photoreceptor outer segments, ingesting choroid-derived lipoproteins, and transporting lipids out of the retina^12^. It is known that RPE cells express canonical pathways involved in lipid homeostasis including the sterol regulatory element-binding protein (SREBF) pathway in mice and humans^27,28^ but may rely on additional mechanisms as suggested by a lack of responsiveness of cholesterol-sensitive genes in wild-type mouse retinas after the consumption of dietary cholesterol^27^. Through our analysis of differential genes that correlated with the number of *Tmem135* mutant alleles, we identified BCOR and IDH1 as potential players in RPE lipid homeostasis. BCOR is a co-repressor of BCL6 that mediates BCL6-mediated transcriptional repression^10^. There have been no published reports on a role for BCOR in lipid metabolism, but loss of BCL6 in the liver augments lipid catabolism, lessens high-fat-diet induced hepatic steatosis, and reverses fatty acid breakdown and lipid accumulation induced by fasting in peroxisome proliferator activated receptor alpha (*Ppara*) knockout mice^29^. The decrease in *Bcor* in the *Tmem135*^*FUN025*^^/*FUN025*^ mouse eyecups may be in response to the increased lipid accumulation such as cholesterol and neutral lipids. IDH1 is an isocitrate dehydrogenase enzyme found in the cytoplasm as well as on peroxisomes that catalyzes the reversible oxidative decarboxylation of isocitrate to alpha-ketoglutarate^30^. Loss-of-function mutations in human *IDH1* lead to increased monosaturated fatty acids and increased SCD expression in infiltrating gliomas^31^. Since there is increased eyecup *Scd1* expression in *Tmem135*^*FUN025*/*FUN025*^ mice and SCD1 is the rate-limiting enzyme for the conversion of saturated fatty acids to monounsaturated fatty acids^32^, the increased *Idh1* expression in *Tmem135*^*FUN025*/*FUN025*^ mouse eyecups may occur to decrease the amount of monounsaturated fatty acids. Further elucidation of BCOR and IDH1 in *Tmem135*^*FUN025*/*FUN025*^ mouse eyecups could lead to insight into lipid metabolism within RPE cells.

Dysregulation of lipid metabolism is strongly associated with AMD development and progression^33^. The association of dysregulated lipid metabolism and AMD comes from genetic and environmental studies. Genetic studies have linked variants with human apolipoprotein E (*APOE*), hepatic lipase (*LIPC*), cholesteryl ester transfer protein (*CETP*), lipoprotein lipase (*LPL*), and ATP binding cassette subfamily A member 1 (*ABCA1*) with risk for AMD^34–40^. The roles of these genes in AMD development and progression are largely unknown. Furthermore, consumption of diets high in fat^41–43^, particularly those with high levels of omega-6, monounsaturated, polyunsaturated and trans unsaturated fatty acids^44,45^, increases the likelihood of developing AMD. In contrast, eating diets with a high concentration of fish oils containing omega-3 fatty acids decreases the incidence of AMD^44–47^. How diets influence retinal homeostasis and modulate the risk for AMD is unclear. To understand the role of these epidemiological risk factors for AMD, it is imperative to study models in which we can gain new insight into AMD disease processes. We have shown in our RNA-Seq study that *Tmem135*^*FUN025*^^/*FUN025*^ mice have altered expression of lipid metabolic genes in their posterior eyecups and lipid accumulation in their RPE. Some of these metabolic genes are commonly changed between *Tmem135*^*FUN025*/*FUN025*^ eyecups and AMD-afflicted RPE/choroid samples. One gene, *Srebf1/SREBF1*, is similarly changed in *Tmem135*^*FUN025*/*FUN025*^ mouse eyecups and RPE/choroid samples from human patients diagnosed with mixed, intermediate, and advanced stages of AMD. SREBF1 is a transcription factor that regulates the expression of sterol-regulated genes and controls cholesterol homeostasis^48^. SREBF1 also positively regulates the expression of lipogenesis genes including *Fasn/FASN*^49^ which is increased in both *Tmem135*^*FUN025*/*FUN025*^ mouse eyecups and RPE/choroid samples from human patients diagnosed with mixed and intermediate AMD. Future studies on the role of SREBF1 in *Tmem135*^*FUN025*/*FUN025*^ retinas may provide clues on its role in AMD development and progression.

Aging is a significant risk factor for a number of diseases including AMD and Alzheimer’s disease, but how aging contributes to these diseases is unknown. One strategy to better understand the role of aging in disease development is to study mutant mice with accelerated aging phenotypes and uncover the genetic causes of their accelerated aging phenotypes. Utilizing this strategy, we identified *Tmem135* as a gene involved in the aging process in the retina^3^. In the current study, we undertook an RNA-Seq study to further investigate the consequences of the *Tmem135* mutation on retinal homeostasis. We found that mutant *Tmem135* mice display significant transcriptomic changes but one significant pathway that contained a number of differentially-expressed genes is lipid metabolism. Some of these genes are also altered in transcriptomic profiles of AMD-diagnosed RPE/choroid specimens. Studying *Tmem135* mutant mice may provide insight into how aging effects lipid metabolism and contributes to diseases such as AMD.

## Methods

### Mice

Generation of *Tmem135*^*FUN025*^^*/*+^ and *Tmem135*^*FUN025/FUN025*^ mice on the C57BL/6 J background was previously described^3,7^. WT mice on the C57BL/6 J background were used as controls for these experiments. All animals were housed in the same animal facility at the University of Wisconsin-Madison under the same environmental conditions. Both male and female mice that were 2.5 months, 3 months and 12 months of age were used in this study. Details of mice used in each figure of this study are provided in Supplemental Table 1. No mice used in this study were positive for the *Ped6b*^*rd*^^1^ or the *Crb1*^*rd*^^8^ mutation^50,51^. All experiments performed in this study were in accordance with the National Institute of Health Guide for the Care and Use of Laboratory Animals and authorized by the Animal Care and Use Committee at the University of Wisconsin-Madison. The results and methods in this study are reported in accordance to the ARRIVE guidelines.

### Histology

Eyes were fixed in 2% PFA and 2% glutaraldehyde overnight at 4 °C and submitted to the University of Wisconsin-Madison's Translational Research Initiatives in Pathology (TRIP) core for paraffin processing. Sections were stained with hematoxylin and eosin (H&E) to visualize retinal layers and imaged using an Axio Imager 2 microscope (Carl Zeiss MicroImaging, White Plains, NY) at a 40X magnification. Outer nuclear layer (ONL) and inner nuclear layer (INL) thickness were measured using ImageJ (NIH, Bethesda, MD) on a single section from each eye. Measurements were started from the optic nerve head and taken at seven consecutive 300 micron intervals.

### Electroretinography (ERG)

Mice were dark-adapted overnight before ERG recording and then anesthetized with a cocktail of ketamine (80 mg/kg) and xylazine (16 mg/kg) diluted in phosphate buffered saline (PBS). Pupils were dilated with 1% tropicamide, and a drop of sterile 2.5% hypromellose ophthalmic solution (Goniovisc, HUB pharmaceuticals LLC, CA) was applied to the cornea for recording electrodes. ERGs were recorded using the Espion system (Diagnosys LLC, MA). Scotopic recordings were obtained from dark-adapted animals, and the eyes were exposed to a sequential increment of flash intensities (0.1 to 30 cd.s/m^2^) for 300 ms with a 2 s interval between the flashes. For the acquisition of c-wave, the eyes were flashed with light intensities of 2.5 and 25 cd.s/m^2^ for 4 s. Photopic recordings were performed after the light adaptation of mice at 30 cd.m^2^ background and during the photopic recordings. The flash intensities used for the photopic recordings were 0.78, 1, 2.25, 5 and 10 cd.s/m^2^. Only the 10 cd.s/m^2^ recordings were used in this study. ERG components were measured using the Espion software (Diagnosys LLC, MA) and analyzed using Origin2020 (OriginLab Corp., MA).

### Bulk-RNA sequencing

Posterior eyecups without the neural retina were collected and pooled from individual 2.5-month-old WT, *Tmem135*^*FUN025*^^/+^, and *Tmem135*^*FUN025*/*FUN025*^ mice of both genders between 11:00 and 1:00 PM. Samples were flash frozen and then submitted to GENEWIZ (South Plainfield, NJ, USA) for processing. Total RNA was extracted from eyecups with an RNeasy Plus Universal Mini kit (Qiagen, Hilden, Germany) following the Manufacturer’s instructions. Total RNA samples were quantified using a Qubit 2.0 Fluorometer (Life Technologies, Carlsbad, CA, USA). RNA integrity was examined using a TapeStation 4200 (Agilent Technologies, Palo Alto, CA, USA). RNA sequencing libraries were prepared using the NEBNext Ultra RNA Library Prep Kit for Illumina (NEB, Ipswich, MA, USA) following manufacturer’s instructions. Briefly, messenger RNAs were first enriched with Oligo(dT) beads. Enriched mRNAs were fragmented for 15 min at 94 °C. First strand and second strand cDNAs were subsequently synthesized. cDNA fragments were end repaired and adenylated at 3’ends, and universal adapters were ligated to cDNA fragments, followed by index addition and library enrichment by limited-cycle PCR. The sequencing libraries were validated on the Agilent TapeStation and quantified by using Qubit 2.0 Fluorometer as well as by quantitative PCR (KAPA Biosystems, Wilmington, MA, USA). The sequencing libraries were clustered on a flowcell. After clustering, the flowcell was loaded on the Illumina HiSeq instrument (4000 or equivalent) according to manufacturer’s instructions. The samples were sequenced using a 2 × 150 bp Paired End (PE) configuration. Image analysis and base calling were conducted by the HiSeq Control Software (HCS). Raw sequence data (.bcl files) generated from Illumina HiSeq were converted into fastq files and de-multiplexed using Illumina's bcl2fastq 2.17 software. The RNA-Seq raw sequence files from this study are available on the Gene Expression Omnibus (GEO), accession number GSE184160.

### RNA sequencing analysis

Gene expression read counts were analysed using NetworkAnalyst 3.0^52^. *M. musculus* organism was selected with bulk sequencing analysis workflow. Quality control step involved filtering genes with very high variance across samples. Genes were ranked based on variance and those genes which ranked in the bottom 15% of the percentile were filtered out. Low abundance genes below a threshold were also filtered out. Data was normalized using log 2 counts per million normalization method. Differential gene expression analysis was performed using EdgeR^53^. Gene set enrichment analysis was performed using WebGestalt^54^ and different functional databases including Gene Ontology, KEGG, and WikiPathways were used for analysis. To further restrict the number of gene sets due to overlap of the genes, affinity cluster algorithm^55^ was applied. Heatmaps were generated on auto-scaled normalized gene expression data using Euclidean distance measure and ward clustering algorithm using MetaboAnalyst 4.0^56^. Signalling pathway analysis was conducted on differentially expressed genes using the SIGNOR 2.0 database^57^. A comparison of differentially expressed genes in different groups with human age-related macular degeneration samples was conducted by comparing the fold change (up or down) of differentially expressed genes identified from integrated microarray (GSE29801 dataset)^17^ and RNA-seq analysis (GSE135092 dataset)^18^ of AMD human donor eyes compared to non-AMD control donor eyes. AMD donor eyes used to generate the GSE29801 dataset were independently classified into distinct AMD categories based on the Age-Related Eye Disease Study (AREDS) and Rotterdam grading scales^17^ whereas AMD donor eyes graded as having category 4 disease were used to produce the GSE135092 dataset^18^. In total, the GSE29801 dataset was comprised of expression data from 31 control eyes and 37 AMD-afflicted eyes^17^ while the GSE135092 dataset contained transcriptomic data from 106 control eyes and 23 AMD eyes^18^.

### Cholesterol measurement

Mice were perfused with PBS prior to enucleation of eyes. Eyes were processed to remove the anterior segment, lens, and neural retina. The remaining posterior eyecups were flash frozen with liquid nitrogen before processing. Frozen eyecups were homogenized using 80 ul of RIPA lysis and extraction buffer (#PI89901, VWR, Radnor, PA) with ethylenediaminetetraacetic acid (EDTA)-free protease inhibitors (#11836170001, Sigma-Aldrich, St. Louis, MO). Cholesterol was measured using a colorimetric Total Cholesterol Assay Kit (#STA-384, Cell Biolabs, San Diego, CA). Cholesterol values were normalized to the weight of the individual eyecup.

### RPE flat mount immunofluorescence

Eyes were removed and processed for RPE flat mounts using a previously described protocol^7^. RPE flat mounts were incubated with a rabbit polyclonal zonula occludens-1 antibody (#40–2200, Thermo Fisher Scientific, Waltham, MA) and HCS Lipidtox Red Neutral Lipid Stain (#H34476, Thermo Fisher Scientific, Waltham, MA) overnight at 4 °C. Then, the eyecups were incubated with an Alexa Fluor 488 conjugated donkey anti-rabbit IgG (#A-21206, Thermo Fisher Scientific, Waltham, MA) and 4′,6-Diamidine-2′-phenylindole dihydrochloride (DAPI) (#D9542, Sigma Aldrich, St. Louis, MO) for two hours. RPE flat mounts were imaged with a Nikon A1RS confocal microscope at a 20× magnification.

### Quantitative real-time PCR

Eyecups and neural retinas from mice were isolated after PBS perfusion, flash frozen, and kept at − 80 °C. RNA was isolated using a RNeasy lipid tissue mini kit (#74804, Qiagen, Germantown, MD). Concentrations of RNA were calculated using a Nanodrop 2000 UV–Vis Spectrophotometer (Thermo Fisher Scientific, Waltham, MA). Equal RNA concentrations were aliquoted to make cDNA using an Oligo d(T)18 primer (#S1316S, NEB, Ipswich, MA) and the ProtoScript II Reverse Transcriptase (#M0368L, NEB, Ipswich, MA). Triplicate reactions for each gene was run in the Roche Lightcycler 480 system using cDNA, 200 nmol/L of each primer, and Lightcycler 480 SYBR Green I Supermix (#507203180, Roche). Primer sequences used in this study are described in Supplemental Table 2. Relative expression was normalized to the ribosomal protein lateral stalk subunit P0 (*Rlpl0*) using the quantitative 2^−ΔΔCT^ method^58^.

### Statistical analysis

Statistics were performed using GraphPad Prism software (GraphPad Software, Inc., San Diego, CA). Details of statistical tests are provided in figure legends.

## Supplementary Information

Supplementary Information.
