# Supplementary material for: A mutation in transmembrane protein 135 impairs lipid metabolism in mouse eyecups
Source: Sci Rep. 2022 Jan 14;12:756. doi: 10.1038/s41598-021-04644-3 (PMC8760256; doi:10.1038/s41598-021-04644-3)
Supplement: Supplementary file 1 — Supplementary Information. [file 41598_2021_4644_MOESM1_ESM.docx]

**Supplemental Table 1: Ages, Genders, and Numbers of Mice Used in Study.**

| \|  \| Panel \| Age (months) \| WT Male \| WT Female \| Total \| *FUN025*/+ Male \| *FUN025*/+ Female \| Total \| *FUN025/FUN025* Male \| *FUN025/FUN025* Female \| Total \| \| --- \| --- \| --- \| --- \| --- \| --- \| --- \| --- \| --- \| --- \| --- \| --- \| \| Fig.1 \| A, B \| 12 \| 3 \| 3 \| 6 \| 2 \| 2 \| 4 \| 2 \| 2 \| 4 \| \| C-G \| 12 \| 3 \| 3 \| 6 \| 4 \| 2 \| 6 \| 2 \| 2 \| 4 \| \| Fig. 2-4 and 7 Tables 1-3 \|  \| 2.5 \| 2 \| 2 \| 4 \| 4 \| 2 \| 6 \| 2 \| 3 \| 5 \| \|  \| \| Fig. 5 \| A \| 3 \| 5 \| 4 \| 9 \| 2 \| 1 \| 3 \| 3 \| 1 \| 4 \|  \| \| B \| 12 \| 2 \| 3 \| 5 \| 3 \| 1 \| 4 \| 3 \| 1 \| 4 \|  \| \| Fig. 6 \| A, B \| 2.5 \| 0 \| 0 \| 0 \| 3 \| 0 \| 3 \| 5 \| 0 \| 5 \|  \| |
| --- | --- | --- | --- | --- | --- | --- | --- | --- | --- | --- | --- | --- | --- | --- | --- | --- | --- | --- | --- | --- | --- | --- | --- | --- | --- | --- | --- | --- | --- | --- | --- | --- | --- | --- | --- | --- | --- | --- | --- | --- | --- | --- | --- | --- | --- | --- | --- | --- | --- | --- | --- | --- | --- | --- | --- | --- | --- | --- | --- | --- | --- | --- | --- | --- | --- | --- | --- | --- | --- | --- | --- | --- | --- | --- | --- | --- | --- | --- | --- | --- | --- | --- | --- | --- | --- | --- |
|  |
|  |

**Supplemental Table 2: List of qPCR Primers**

| Gene | Forward Primer | Reverse Primer |
| --- | --- | --- |
| *Srebf2* | GCAGCAACGGGACCATTCT | CCCCATGACTAAGTCCTTCAACT |
| *Srebf1c* | TCAAAACCAGCCTCCCAAGA | CCCCGTCCACAAAGAAACG |
| *Acaca* | TGTACAAGCAGTGTGGGCTGGCT | CCACATGGCCTGGCTTGGAGGG |
| *Fasn* | GCTGCGGAAACTTCAGGAAAT | AGAGACGTGTCACTCCTGGACTT |
| *Scd1* | TTCTTGCGATACACTCTGGTGC | CGGGATTGAATGTTCTTGTCGT |
| *Rplp0* | GGACCCGAGAAGACCTCCTT | GCACATCACTCAGAATTTCAATGG |

|  |  |  |  |  |  |  |  |  |  |  |  |  |
| --- | --- | --- | --- | --- | --- | --- | --- | --- | --- | --- | --- | --- |
|  |  |  |  |  |  |  |  |  |  |  |  |  |
|  |  |  |  |  |  |  |  |  |  |  |  |  |
|  |  |  |  |  |  |  |  |  |  |  |  |  |
|  |  |  |  |  |  |  |  |  |  |  |  |  |
|  |  |  |  |  |  |  |  |  |  |  |  |  |
|  |  |  |  |  |  |  |  |  |  |  |  |  |
|  |  |  |  |  |  |  |  |  |  |  |  |  |
|  |  |  |  |  |  |  |  |  |  |  |  |  |
